# Fasudil attenuates aggregation of α-synuclein in models of Parkinson’s disease

**DOI:** 10.1186/s40478-016-0310-y

**Published:** 2016-04-22

**Authors:** Lars Tatenhorst, Katrin Eckermann, Vivian Dambeck, Luis Fonseca-Ornelas, Hagen Walle, Tomás Lopes da Fonseca, Jan C. Koch, Stefan Becker, Lars Tönges, Mathias Bähr, Tiago F. Outeiro, Markus Zweckstetter, Paul Lingor

**Affiliations:** Deparment of Neurology, University Medicine Göttingen, Robert-Koch-Str. 40, 37075 Göttingen, Germany; Max Planck Institute for Biophysical Chemistry, Am Fassberg 11, 37077 Göttingen, Germany; Deparment of NeuroDegeneration and Restorative Research, University Medicine Göttingen, Waldweg 33, 37073 Göttingen, Germany; Instituto de Fisiologia, Faculty of Medicine, University of Lisbon, Lisboa, 1649-028 Portugal; DFG Research Center for Nanoscale Microscopy and Molecular Physiology of the Brain (CNMPB), Humboldtalle 23, 37073 Göttingen, Germany; German Center for Neurodegenerative Diseases (DZNE), Am Fassberg 11, 37077 Göttingen, Germany; Current address: Neurological Clinic, St. Josef-Hospital, Ruhr-University Bochum, Gudrunstr. 56, 44791 Bochum, Germany

**Keywords:** α-synuclein aggregation, Fasudil, Parkinson’s disease, A53T mouse model

## Abstract

Parkinson’s disease (PD) is the most common neurodegenerative movement disorder, yet disease-modifying treatments do not currently exist. Rho-associated protein kinase (ROCK) was recently described as a novel neuroprotective target in PD. Since alpha-synuclein (α-Syn) aggregation is a major hallmark in the pathogenesis of PD, we aimed to evaluate the anti-aggregative potential of pharmacological ROCK inhibition using the isoquinoline derivative Fasudil, a small molecule inhibitor already approved for clinical use in humans. Fasudil treatment significantly reduced α-Syn aggregation *in vitro* in a H4 cell culture model as well as in a cell-free assay. Nuclear magnetic resonance spectroscopy analysis revealed a direct binding of Fasudil to tyrosine residues Y133 and Y136 in the C-terminal region of α-Syn. Importantly, this binding was shown to be biologically relevant using site-directed mutagenesis of these residues in the cell culture model. Furthermore, we evaluated the impact of long-term Fasudil treatment on α-Syn pathology *in vivo* in a transgenic mouse model overexpressing human α-Syn bearing the A53T mutation (α-Syn^A53T^ mice). Fasudil treatment improved motor and cognitive functions in α-Syn^A53T^ mice as determined by Catwalk^TM^ gait analysis and novel object recognition (NOR), without apparent side effects. Finally, immunohistochemical analysis revealed a significant reduction of α-Syn pathology in the midbrain of α-Syn^A53T^ mice after Fasudil treatment. Our results demonstrate that Fasudil, next to its effects mediated by ROCK-inhibition, directly interacts with α-Syn and attenuates α-Syn pathology. This underscores the translational potential of Fasudil as a disease-modifying drug for the treatment of PD and other synucleinopathies.

**Electronic supplementary material:**

The online version of this article (doi:10.1186/s40478-016-0310-y) contains supplementary material, which is available to authorized users.

## Introduction

In the coming decades the number of patients affected by neurodegenerative disorders, including Parkinson’s disease (PD), will inevitably increase [12]. Neurodegeneration in PD is accompanied by the appearance of Lewy bodies, intra-neuronal protein aggregates with α-synuclein (α-Syn) as major component [4]. Initially established as histopathological marker for PD, aggregated α-Syn is now regarded as key pathogenic agent [4]. Although α-Syn is a relatively small molecule of 140 amino acids and can access different cellular compartments [66], it is predominantly located in presynaptic terminals, associated to membranous structures like synaptic vesicles [4]. The discovery that point mutations in α-Syn like A53T or A30P as well as *α-Syn* gene multiplications facilitate the development of PD, underlines the importance of α-Syn in this disease [28, 47, 52]. Furthermore, α-Syn pathology is not restricted to dopaminergic cells, but was also identified in neuronal cell populations of the enteric nervous system and olfactory bulb, explaining the variety of non-motor symptoms in PD [48]. Several animal models using α-Syn mutants were generated, either virus-mediated [38], or using transgenic approaches. Transgenic mouse lines expressing human α-Syn-A30P or -A53T develop motor and cognitive impairments, being in part similar to human PD symptoms, along with nigrostriatal degeneration and aggregate pathology [16–19, 31, 40].

In previous studies in cell culture and animal models of PD, Rho-associated protein kinase (ROCK) was identified as novel molecular neuroprotective target [27, 50, 58, 59, 61]. Pharmacological ROCK inhibition protected against MPTP toxicity, attenuating dopaminergic cell death and increasing regenerative sprouting *in vivo* [59]. Since α-Syn aggregation is a major step in the pathogenesis of PD, we now evaluated the anti-aggregative potential of pharmacological ROCK inhibition using the isoquinoline derivative Fasudil, a small molecule inhibitor already approved for clinical use in humans in Japan [44].

In a cell culture model in H4 human neuroglioma cells [42], we studied the effects of Fasudil treatment on α-Syn aggregation. In a cell-free aggregation assay the direct interaction of Fasudil and α-Syn was investigated and nuclear magnetic resonance (NMR) spectroscopy was then used to identify the binding sites of Fasudil on α-Syn. Finally, a long-term treatment study in the α-Syn^A53T^ mouse model [16] was performed to investigate the impact of Fasudil application on α-Syn pathology *in vivo*. Our results identify Fasudil as a drug with high translational potential as disease-modifying treatment for PD and other synucleinopathies.

## Materials and methods

### Cell free α-Syn aggregation assay

Recombinant α-Syn was expressed and purified as described previously [22]. Samples containing α-Syn were diluted to a concentration of 100 μM in a 50 mM HEPES (Sigma, Taufkirchen, Germany) buffer with 100 mM NaCl (Sigma) and 0.03 % NaN_3_ (Sigma) at pH 7.4 in a 500 μl total volume and subsequently subjected to aggregation-prone conditions (37 °C, ~300 rpm) in low binding protein 1.5 ml Eppendorf tubes during at least 14 days in the absence and presence of increasing concentrations of Fasudil (LC Laboratories, Woburn, MA) and Y-27632 (Tocris Bioscience, Bristol, UK) corresponding to compound to protein ratios from 1:1 to 40:1. At different incubation times, a 10 μl aliquot of the sample was mixed with 1 ml of a 100 mM ThT (Sigma) solution. Its fluorescence emission was measured between 460 and 600 nm on a Varian Cary Eclipse fluorescence spectrophotometer (Agilent Technologies, Santa Clara, CA) with excitation wavelength of 442 nm at 20 °C and with a 5 nm excitation and emission slit. The averaged intensity values of the maximum peak (~480 nm) were fitted to a sigmoidal equation and presented as a function of time.

### Electron microscopy of α-Syn fibrils

A solution containing protein was applied to glow-discharged carbon coated grids and stained with 1 % uranyl acetate (Sigma). Images were taken in a Philips CM120 electron microscope (Philips, Amsterdam, The Netherlands) at a defocus of 2.3 μm using a TemCam 224A slow scan CCD camera (TVIPS, Gauting, Germany).

### NMR spectroscopy of α-Syn

Recombinant ^15^N-labeled α-Syn was expressed and purified as described previously [22]. NMR samples contained 0.1 mM ^15^N-labeled wild-type (wt) α-Syn in 50 mM HEPES buffer, 100 mM NaCl, pH 7.4 and 90 % H_2_O/10 % D_2_O. NMR experiments were recorded on a Bruker Avance 600 MHz spectrometer. The temperature was set to 20 °C. Data processing was performed using the software packages Topspin (Bruker, Billerica, MA) and CCPN Analysis [64]. ^15^N-^1^H heteronuclear single quantum coherence (HSQC) amide cross-peaks affected during compound addition were identified by comparison of their chemical shift values with those of the same cross-peaks in the data set of samples lacking the compound. Perturbations in the chemical shift values for ^1^H and ^15^N were calculated as [(Δδ^1^H)^2^ + (Δδ^15^N/10)^2^]^1/2^.

### Plasmids

To investigate the impact of Fasudil treatment on α-Syn aggregation, we used an aggregation model with co-transfection of synphilin-1 (SYPH1) and carboxy-terminally truncated αSyn.EGFP fusion protein (synT) in H4 human neuroglioma cells *in vitro*, leading to the formation of intracellular SYPH1/synT inclusions within 24 h after transfection. The corresponding plasmids pcDNA3.1/synT and pcDNA3.1/V5-SYPH1 were described before [42].

Mutants of synT were generated by site-directed mutagenesis using the following primers (5'-3'):

- Y133A fw AAATGCCTTCTGAGGAAGGGGCTCAAGACTACGAACCTGAA,
- rev CTTCAGGTTCGTAGTCTTGAGCCCCTTCCTCAGAAGGCATTT;
- Y136A fw TTCTGAGGAAGGGTATCAAGACGCCGAACCTGAAGCCGGTA,
- rev CCCGCGGTACCGGCTTCAGGTTCGGCGTCTTGATACCCTT;
- Y133A/Y136A fw AAATGCCTTCTGAGGAAGGGGCTCAAGACGCCGAACCTGAAG, rev TACCGGCTTCAGGTTCGGCGTCTTGAGCCCCTTCCTCAGAA.

### Antibodies

The following primary antibodies were used: mouse anti-α-Syn (BD610787, BD Biosciences, Franklin Lakes, NJ), rabbit anti-V5 (ab9116, Abcam, Cambridge, UK), mouse anti-α-Syn (LB509, Covance, Munich, Germany), rabbit anti-TH (Zytomed, Berlin, Germany), rabbit anti-TPH2 (ABN60, Millipore, Darmstadt, Germany), mouse anti-NeuN (MAB377, Millipore) and mouse anti-β-tubulin (Sigma, St- Louis, MO).

The following secondary antibodies were used: Cy2-conjugated goat anti-mouse, Cy3-conjugated goat anti-rabbit, Cy3-conjugated goat anti-mouse, Cy5-conjugated goat anti-rabbit (all from Jackson Immuno Research, West Grove, PA); horseradish peroxidase(HRP)-coupled goat anti-mouse and HRP-coupled goat anti-rabbit (both from Dianova, Hamburg, Germany).

### Treatment, transfection, and immunocytochemistry of H4 cells

H4 human neuroglioma cells (HTB-148, ATCC, Manassas, VA) were plated on poly-D-lysine (Sigma)-coated glass cover slips at a density of 35,000/cm^2^ and pre-incubated with 5 and 20 μM Fasudil or Y-27632 in DMEM (Gibco, Karlsruhe, Germany) supplemented with 10 % fetal bovine serum (PAA Laboratories, Pasching, Germany), 100 U/ml Penicillin (Sigma), and 100 μg/ml Streptomycin (Sigma). 24 h later, cells were transfected with FuGENE HD (Promega, Madison, WI) using wt or mutant synT and SYPH1 plasmid DNA (total DNA 0.25 μg/cm^2^), and incubated for additional 24 h with Fasudil or Y-27632. After washing with PBS (Applichem, Darmstadt, Germany), cells were fixed in PBS/4 % paraformaldehyde (PFA, Applichem) for 10 min, permeabilized in PBS/0.5 % Triton X-100 (Applichem) for 20 min, and blocked in PBS/1.5 % normal goat serum (NGS; PAA) for 1 h. Cells were incubated with primary antibodies (mouse anti-α-Syn, 1:1000; rabbit anti-V5, 1:5000) at 4 °C over night, washed in PBS and treated with respective secondary antibodies (1:250) for 2 h followed by staining with 2 μg/ml 4',6'-Diamidin-2-phenylindol (Merck, Darmstadt, Germany) for 3 min and mounting in Fluoromount G (Southern Biotech, Birmingham, AL). Images were acquired using an Axioplan2 equipped with AxioCam HRm camera and AxioVision SE64 Rel. 4.9 software (Carl Zeiss, Göttingen, Germany). Transfected cells with and without inclusions from 9 images per experimental conditions of at least three independent experiments were counted and compared between groups.

### Western blot analysis of H4 cells

H4 cells were lysed 24 h after transfection in RIPA buffer consisting of 20 mM sodium phosphate (pH7.4; Roth, Karlsruhe, Germany), 150 mM NaCl (Roth), 1 % Triton X-100 (Roth), 0.5 % sodium deoxycholate (Sigma), 0.5 % SDS (Roth), and protease inhibitors (‘Complete tablets’, Roche, Basel, Switzerland). Briefly, protein content of the samples was determined by BCA assay (ThermoScientific, Rockford, IL, USA), and equal amounts of protein (20 μg) were separated on a sodium dodecyl sulfate polyacrylamide gel electrophoresis (SDS-PAGE) and blotted onto a nitrocellulose membrane (Applichem). After blocking with 5 % nonfat milk (Applichem) in Tris-buffered saline/Tween 20 (TBS-T, Applichem) for 1 h membranes were incubated with respective primary antibodies for 18 h at 4 °C in TBS-T and 5 % milk. Finally, membranes were incubated with corresponding horseradish peroxidase-coupled secondary antibodies (1:4000 for 1 h at room temperature) and chemiluminescence signal was visualized and quantified using ECL (Immobilon Western, Millipore, Billerica, MA, USA) and ChemiDoc XRS+ with Image Lab Software (BIO-RAD, Hercules, CA, USA).

### SEC-HPLC and dotblot analysis

H4 neuroglioma cells were collected 24 h after transfection in a phosphate buffer (1X PBS with 0.5 % TritonX-100) freshly supplemented with protease inhibitor cocktail (Roche, Mannheim, Germany) and centrifuged for 10 min at 10,000 g. 2-3 mg of total protein in a maximum volume of 500 μl was filtered using a 0.45 μm Spin-X centrifuge filter before loading onto a Superose 6 (Superose 6 10/300GL. GE Healthcare Life Science, Sweden) column and subsequent high-performance liquid chromatography (HPLC) (Äkta Purifier 10, GE Healthcare). The run was performed with a flow rate of 0.5 ml/min and fractions of 500 μl were collected. For the dot blot assay, these fractions were boiled at 95 °C for 10 min and centrifuged at 10,000 g for 5 min. Afterwards they were loaded to a nitrocellulose membrane with the following procedure being identical to the previously described Western blot method.

### Animal experiments

Animals were treated according to the regulations of the local animal research council and legislation of the State of Lower Saxony, Germany (33.19-42502-04-12/0884). Heterozygous breeding couples of the transgenic mouse strain B6;C3-Tg(Prnp-SNCA*A53T)83Vle/J (short: α-Syn^A53T^) were bought from Jax Labs (J004479; Bar Harbor, ME) and maintained at the Central Animal Care Unit of the University Medicine Göttingen, Germany. Offspring were genotyped using a qPCR protocol according to Jax Labs’ instructions to discriminate homozygous, heterozygous and wt animals. We selected homozygous transgenic mice and wt littermates for our experiments. Mice were housed in groups of five in individually ventilated cages (IVC, Tecniplast, Hohenpeißenberg, Germany) under a 12 h light/12 h dark cycle with free access to food and water. To monitor for changes in weight, all mice were weighed once a week. From day 50 on mice were treated with Fasudil at a dosage of 10 mg or 30 mg/kg bodyweight (bw) via the drinking water. A daily drinking amount of 6 ml per animal was taken as a basis and closely monitored to ensure an adequate dosage as described before [58–60]. Treatment groups were as follows: wildtype control (wt ctrl), α-Syn^A53T^ untreated control (A53T ctrl), α-Syn^A53T^ mice treated with 10 mg/kg bw (A53T Fas10) and α-Syn^A53T^ mice treated with 30 mg/kg bw (A53T Fas30). To assess possible acute effects of Fasudil treatment, a separate cohort of α-Syn^A53T^ mice was treated with 30 mg/kg bw Fasudil in the drinking water for 24 h, or with 20 mg/kg bw Fasudil via oral gavage. Before each treatment, α-Syn^A53T^ mice were tested on rotarod and Catwalk to obtain a basic score. After 24 h Fasudil treatment via drinking water, or 30 min after oral gavage, α-Syn^A53T^ mice were tested again on rotarod and Catwalk, and the results obtained were compared to baseline scores.

Blood of randomly selected mice was analyzed to monitor for alterations of standard clinical chemistry and hematology parameters due to Fasudil treatment. Approximately 200 μl blood were collected for hematological analysis from the retrobulbar venous plexus and transferred to ethylene diamine tetra-acetic acid (EDTA) tubes, the age of mice ranged from 200 to 700 days. For biochemical analysis, about 1 ml blood was collected under deep anesthesia with ketamine 200 mg/kg bw (Medistar, Ascheberg, Germany) and xylazine 10 mg/kg bw (Ecuphar, Greifswald, Germany) by heart puncture and transferred to heparin tubes, mice were sacrificed immediately afterwards. Here, the age of mice ranged from 100 to 700 days. Blood samples were analyzed in the core facility of the Clinical Chemistry Department of the University Medicine Göttingen, Germany. Since α-Syn^A53T^ mice develop a severe movement disorder starting with a paresis of the hind limbs, earliest onset after about 8 months of age [16], mice were closely monitored from day 220 on regarding weight and motor behavior on the rotarod. After clinical symptoms manifested, mice underwent for the last time additional behavioral tests as described below. To prevent animals from suffering further paresis, mice were sacrificed after behavioral tests were performed.

### Rotarod

To measure motor balance and coordination, mice were tested on an accelerated rotarod (rotarod for mice 47600, Ugo Basile, Comerio, Italy) ranging from 5 to 40 rpm within 5 min as described before [58, 59]. All mice were pre-trained on the rotarod in order to reach a stable performance. Rotarod test was performed after 100 d, 200 d, and from day 220 on twice a week, each time in three sessions with an intertrial interval of 30 min to reduce stress and fatigue. Rotarod performance in all three runs was recorded and the average time on the rotarod was evaluated. The continuous rotarod testing twice a week from day 220 on was used to detect the onset of clinical pathology described for this mouse model [16] as early as possible for each animal. When rotarod performance of a single mouse dropped below 50 % of its long term average, the respective animal was termed as ‘disease onset’ and ultimately tested in Catwalk gait analysis and novel object recognition test.

### Catwalk gait analysis

Catwalk XT gait analysis system (Noldus, Wageningen, The Netherlands) was used to monitor gait performance of α-Syn^A53T^ mice and wt controls as described before [50]. To address possible changes in gait performance during disease progression, mice were tested before possible clinical symptoms at 200 days, as well as at ‘disease onset’. During the test animals were placed in a walkway of 4 cm width and videotaped from below. Footprints were automatically detected by the Catwalk XT 10.0 software. Detection settings were as follows: camera gain 20; intensity threshold 0.10; max. allowed speed variation 60 %. Three compliant runs per animal were recorded and means out of these runs were analyzed over treatment groups with Catwalk XT 10.0 gait analysis software.

### Novel object recognition test

To monitor for cognitive deficits in α-Syn^A53T^ mice, the novel object recognition (NOR) test was performed as described before [6]. Briefly, single mice were placed in an arena of 48 × 35 cm for 3 min to habituate, and then two identical objects were put into the arena to be explored by the mouse for 5 min to familiarize. After a 10 min recovery in the home cage, mice were put back into the arena for 5 min, where one of the familiar objects had been changed to a novel one, which the mouse had never seen before. During the test mice were videotaped, and the time spent with familiar and novel object, as well as general movement parameters (mobility, velocity, distance moved) were detected by Ethovision XT 8.5 software (Noldus; Fig 6a, b). The discrimination ratio (time spent with novel object/time spent with both objects) was calculated as described before [6] and compared between the treatment groups.

### Immunohistochemistry

Mice were sacrificed by exposure to carbon dioxide and directly afterwards perfused transcardially with PBS, followed by 4 % paraformaldehyde (PFA; Applichem) in PBS (pH 7.4). Brains were dissected, post-fixed in PFA overnight at 4 °C, and finally embedded into paraffin. Brains were coronally sectioned into 5 μm slices, mounted on slides and deparaffinized by xylene (Sigma) treatment for 2x 10 min, followed by a descending ethanol (EtOH) row (2x 3 min 100 % EtOH (Applichem), 96 %, 90 %, 70 %, 50 % EtOH for 3 min each). For immunohistochemistry, sections were incubated for 30 min in citrate buffer (10 mM citric acid (Sigma), 0.05 % Tween 20 (Applichem), pH 6.0 at 80 °C. After cooling down, sections were either treated with 10 μg/ml proteinase K (PK, Applichem) in PBS at 55 °C for 12 min to quantify α-Syn pathology, or just washed with PBS, followed by incubation in 0.1 g sudan black (Applichem) per 100 ml 70 % EtOH. After rinsing with water and PBS washing, sections were incubated for 20 min in 25 mM glycine (Applichem) in PBS. Afterwards sections were blocked for 1.5 h in a solution of 10 % normal horse serum (NHS, PAA), 5 % bovine serum albumin (BSA, Applichem), 0.3 % Triton-X 100 (Applichem) and 25 mM glycine in PBS. First antibodies against α-Syn (1:500, Covance), TH (1:1000, Zytomed), TPH2 (1:100, Millipore) or NeuN (1:100, Millipore) diluted in blocking solution were applied to the sections and incubated in a wet chamber over night at 4 °C. After 3x washing in PBS respective secondary antibodies were used (goat anti-mouse Cy3, 1:250; goat anti-rabbit Cy5, 1:250) and incubated for 1.5 h at room temperature. After 3x washing in PBS, cell nuclei were counterstained with DAPI (Sigma) for 2 min, after final washing in PBS sections were dried and embedded with Mowiol (Sigma). From each brain region, namely red nucleus (RN), substantia nigra (SN) and dorsal raphe nucleus (DR), three to five sections were stained and evaluated by a blinded investigator. Cells with proteinase-K resistant α-Syn aggregates as a marker for α-Syn pathology, as well as neuronal cells were counted in the respective brain regions. The means of at least three independent stainings were calculated and normalized to the respective region’s area as determined by immunohistochemical staining against NeuN for the RN, TH for the SN and TPH2 for the DR.

### Statistical analysis

Statistical analyses were conducted using Kyplot software (Version 2.0, KyensLab Incorporated, Tokyo, Japan). Comparisons of two groups were done by unpaired Student’s *T*-Test, multiple group comparisons by one-way ANOVA with Dunnett post-hoc test. The statistical test and number of *in vitro* experiments or animals used for analysis is indicated in each figure legend. Data are presented as mean ± SD or mean ± SEM, as indicated. Differences were considered significant when *p* < 0.05 (*p < 0.05; **p < 0.01; ***p < 0.001; n.s. = not significant).

## Results

### Fasudil alters synT aggregation in the H4 aggregation model

**Fig. 1:**
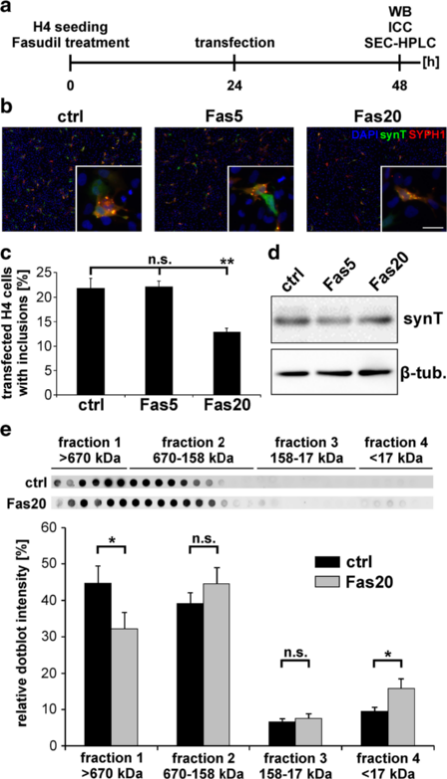
Fasudil treatment significantly reduces synT aggregation in H4 cells *in vitro*. **a** H4 neuroglioma cells were seeded in the presence or absence of Fasudil and 24 h later transfected with plasmids encoding for synT and SYPH1. 24 h after transfection, cultures were investigated by immunocytochemistry (ICC), Western blot (WB) and size-exclusion-chromatography-HPLC (SEC-HPLC). **b** ICC of synT and SYPH1 in H4 cells 24 h after transfection, treated with different Fasudil concentrations. Scale bar: 50 μm. **c** Quantification of transfected H4 cells with inclusions 24 h after transfection and treatment with different Fasudil concentrations. **p < 0.01, ANOVA with Dunnett post-hoc test, *n* = 4. **d** WB analysis of H4 protein lysates 24 h after transfection treated with different Fasudil concentrations revealed no significant differences in protein levels between groups. **e** Quantification of dot-blot analysis against α-Syn after SEC-HPLC fractionation of H4 protein lysates 24 h after Fasudil treatment. Fractions were grouped according to gel filtration standard components. Fraction 1: >670 kDa, fraction 2: 670-158 kDa, fraction 3: 158-17 kDa, fraction 4: <17 kDa. Data are given as mean ± SEM. *p < 0.05, *T*-Test, n(ctr): 8; n(Fas20): 6

Co-transfection of synT and SYPH1 leads to formation of intracellular inclusions within 24 h after transfection [30, 42]. Treatment with Fasudil, beginning 24 h before transfection, reduced the number of transfected H4 cells with inclusions in a dose-dependent manner within 24 h (Fig. 1a, b).

As compared to untreated control (ctrl: 21.8 ± 1.95 %), treatment with 20 μM Fasudil significantly reduced the percentage of cells with inclusions by almost 50 % (Fas20: 12.8 ± 0.82 %, *p* = 0.007, ANOVA with Dunnett post-hoc test) (Fig. 1c). Western blot analysis of protein lysates collected 24 h after transfection showed that Fasudil treatment did not significantly change synT protein levels (Fig. 1d). In comparison, Y-27632 treatment did not have any significant effect on the number of transfected H4 cells with inclusions (Additional file 1: Figure S1). Therefore, a concentration of 20 μM Fasudil was used in the ongoing cell culture experiments.

SEC-HPLC was used to separate protein fractions according to size with regard to gel filtration standard components. Protein fractions were collected and analyzed by dot-blot against synT. Fasudil treatment significantly decreased the number of high molecular weight aggregates, and simultaneously increased the protein amount in the fraction <17 kDa (fraction 1 > 670 kDa: 44.7 ± 4.62 % vs. 32.1 ± 4.55 %, *p* = 0.02, *T*-Test; fraction 2 670-158 kDa: 39.1 ± 2.93 % vs. 44.6 ± 4.43 %; fraction 3 158-17 kDa: 6.6 ± 0.76 % vs. 7.5 ± 1.32 %; fraction 4 < 17 kDa: 9.5 ± 1.01 % vs. 15.7 ± 2.68 %, *p* = 0.03, *T*-Test), possibly suggesting a shift of aggregated synT to monomeric synT after Fasudil treatment (Fig. 1e).

### Fasudil attenuates α-Syn aggregation by direct C-terminal interaction

**Fig. 2:**
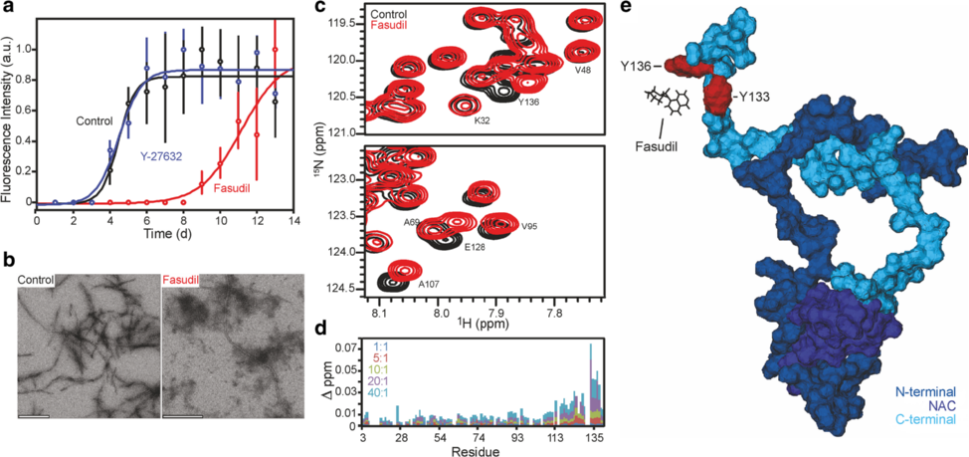
Fasudil delays the aggregation of α-Syn in solution through interaction with the α-Syn C-terminal domain. **a** ThT aggregation monitoring of a 100 μM solution of α-Syn shows a longer nucleation phase and a smaller slope in presence of a 10:1 Fasudil to protein ratio. Data are given as mean ± SD of at least three independent experiments, *p* < 0.001, ANOVA with Dunnett post-hoc test. Note that the ROCK inhibitor Y-27632 has no effect on the aggregation kinetics even in the same concentration. **b** Electron micrographs show a decreased fibril formation after 10 days of incubation in aggregation-prone conditions when Fasudil is present. Scale bars represent 200 nm. **c** Selected regions of 2D ^15^N-^1^H HSQC spectra of α-Syn in the absence (black) and presence (red) of a 20:1 Fasudil to protein ratio. **d** Chemical shift perturbation plot of ^15^N/^1^H resonances of α-Syn in the presence of increasing concentrations of Fasudil (Fasudil-to-α-Syn molar ratios are indicated). Note that the most affected amino acid residues are in the C-terminal region, especially Y133 and Y136. **e** Structural representation of α-Syn and Fasudil’s interaction through the C-terminal region (light blue), specifically in residues Y133 and Y136 (red). The N-terminal region of α-Syn is indicated in dark blue, the non-Aβ component (NAC) domain is marked purple

α-Syn is a natively unfolded protein that upon exposure to aggregation-prone conditions forms amyloid fibrils that are reactive with Thioflavin T (ThT) and are visible by electron microscopy (EM). To probe the influence of Fasudil on the aggregation kinetics of α-Syn, we monitored the change in ThT fluorescence of samples both in the presence and absence of Fasudil as well as the structurally unrelated ROCK-inhibitor Y-27632, a 4-aminopyridine derivative (Fig. 2a). The data show a clear delay in α-Syn aggregation in presence of Fasudil, increasing the lag phase from ~2.5 to ~8 days. Comparison of Fasudil (Fas) to control (ctrl) after 9 days revealed a significant difference (ctrl: 1.01 ± 0.16, *n* = 3; Fas: 0.12 ± 0.06; *n* = 3, *p* = 0.0002, ANOVA with Dunnett post-hoc test), while the ROCK inhibitor Y-27632 had no effect on α-Syn aggregation in this assay (Y-27632: 0.95 ± 0.13, *n* = 3, *p* = 0.79, ANOVA with Dunnett post-hoc test; Fig. 2a). It is worth noting that the slope of the curve associated with elongation of the fibril was steeper in the control and Y-27632 samples as compared to Fasudil, suggesting that Fasudil not only affects formation of aggregation seeds but also the elongation of amyloid fibrils of α-Syn. The lack of defined amyloid fibrils after 10 days of aggregation in the presence of Fasudil was further corroborated by EM (Fig. 2b), as opposed to the classical mature fibrillar structures formed in the absence of Fasudil.

In order to obtain insight into the molecular nature of the interaction between α-Syn and Fasudil, we took advantage of solution nuclear magnetic resonance (NMR) analysis. NMR resonances are a reflection of the microenvironment in which individual amino acids are found and, therefore, are highly sensitive probes of protein-protein and/or protein-ligand interactions, allowing a detailed description of interaction interfaces. Here, we made use of two-dimensional ^15^N-^1^H HSQC experiments in the presence of increasing amounts of Fasudil (Fig. 2c). The perturbation in the relative position of signals in the NMR spectra allowed us to determine that Fasudil preferentially interacts with the C-terminal region of α-Syn, especially with the aromatic residues Y133 and Y136, as determined by the large chemical shift perturbation of their NMR resonances (Fig. 2d). A structural representation of α-Syn and Fasudil’s interaction through the C-terminal region, specifically in residues Y133 and Y136, is given in Fig. 2e. The α-Syn model was taken from a molecular dynamics simulation [46] with Fasudil placed manually in the proximity of residues Y133 and Y136 with the molecular visualization software VMD [24].

### Effect of Fasudil on SynT aggregation is attenuated after Y133/136A substitution

**Fig. 3:**
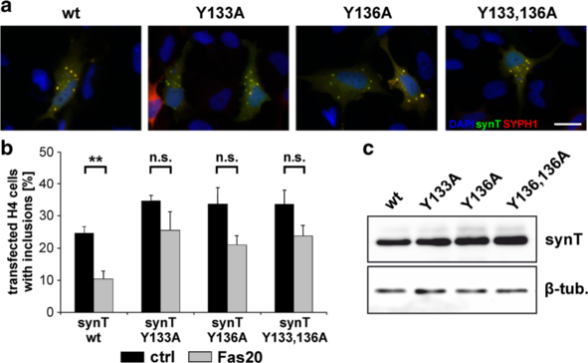
Substitution of Y133 and Y136 with alanine in synT blocks the aggregation-inhibiting effect of Fasudil. **a** H4 neuroglioma cells were treated with 20 μM Fasudil or vehicle and transfected with wt synT or the indicated mutants together with SYPH1. 24 h post-transfection, cells were fixed and immunostained against synT and SYPH1. Representative images show the expression of synT, SYPH1 and DAPI positive nuclei in the absence of Fasudil. Scale bar 25 μm. **b** Transfected cells with and without inclusions from 9 images per experimental conditions of *n* = 4 independent experiments were counted. Data are given as mean ± S.E.M, **p < 0.01, n.s. = not significant, *T*-Test. **c** Western blot analysis of H4 protein lysates from wt or indicated mutants 24 h after transfection revealed no significant changes in synT protein expression

To address the influence of Y133 and Y136 on α-Syn aggregation in the H4 cell culture model *in vitro*, both residues were substituted with alanine in human synT by site-directed mutagenesis, either as single (synT-Y133A and synT-Y136A) or double mutant (synT-Y133,136A). Number, size and distribution of inclusions in single H4 cells were not affected after transfection with the respective mutant synT as compared to wildtype (wt) synT (Fig. 3a).

Again, treatment of H4 cells with 20 μM Fasudil significantly reduced the number of transfected cells with inclusions in case of wt synT (24.6 ± 1.93 % vs. 10.4 ± 2.37 %, *p* = 0.007, *T*-Test), but had no significant effect on the mutants synT-Y133A (34.7 ± 1.66 % vs. 25.6 ± 5.63 %), synT-Y136A (33.7 ± 5.08 % vs. 21.0 ± 2.89 %), or the double mutant synT-Y133A/Y136A (33.5 ± 4.44 % vs.23.8 ± 3.20 %), indicating that synT aggregation can be impaired by Fasudil via interaction with the C-terminal tyrosine residues 133 and 136 (Fig. 3b).

Western blot analysis of synT, normalized to β-tubulin, revealed that neither the single mutations synT-Y133A (1.83 ± 0.08) and synT-Y136A (2.75 ± 0.72), nor the double mutant synT-Y133/136A (2.27 ± 0.92) influenced synT expression on the protein level as compared to wt synT (0.98 ± 0.03) (data given as means ± SEM, *n* = 3 independent experiments, not significant, ANOVA) (Fig. 3c).

### Effects of long-term treatment of α-Syn^A53T^ mice with Fasudil

**Fig. 4:**
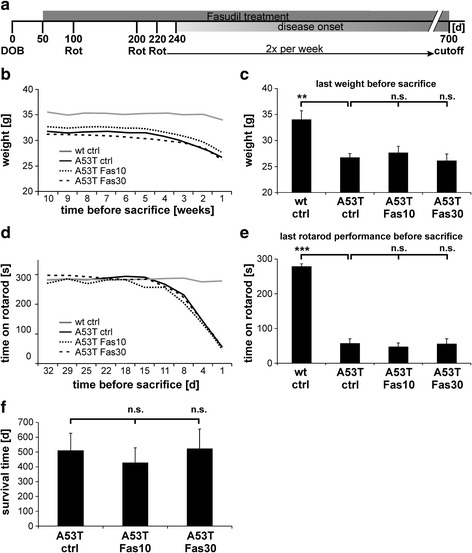
Fasudil treatment does not affect weight, rotarod performance or overall survival of α-Syn^A53T^ mice. **a** 50 days after birth (DOB = day of birth) of α-Syn^A53T^ mice, Fasudil treatment with 10 mg/kg bw and 30 mg/kg bw was started. After 100 and 200 days, mice were tested on a rotarod (Rot). From day 220 on, rotarod was performed twice a week to monitor for onset of clinical symptoms of α-Syn^A53T^ mice. **b, c** Weight of α-Syn^A53T^ mice was monitored weekly. The last 10 weight measurements are displayed (**b**), as well as last weight at day of sacrifice (**c**). **d, e** Rotarod analysis of α-Syn^A53T^ mice treated with 10 mg/kg bw and 30 mg/kg bw Fasudil as compared to untreated α-Syn^A53T^ controls and wildtype controls. The last 10 rotarod performances before sacrifice are displayed for all animals in the respective treatment groups (**d**), as well as the last rotarod performance before sacrifice, showing no significant differences between α-Syn^A53T^ mice with regard to Fasudil treatment (**e**). **f** Survival of α-Syn^A53T^ mice treated with 10 mg/kg bw and 30 mg/kg bw Fasudil as compared to untreated α-Syn^A53T^ controls, showing no significant differences between the groups. All data are given as means ± SEM; n(wt ctrl) = 10, n(A53T ctrl) = 10, n(A53T Fas10) = 10, n(A53T Fas30) = 9; **p < 0.01, ***p < 0.001, n.s. = not significant; *T*-Test for comparisons between ctrl groups; ANOVA with Dunnett post-hoc test for multiple group comparisons between α-Syn^A53T^ groups

To investigate the influence of long-term Fasudil treatment on α-Syn pathology *in vivo* we used a mouse model expressing human α-Syn^A53T^ under the prion protein (Prnp) promoter [16]. Homozygous α-Syn^A53T^ mice develop a neuronal α-synucleinopathy with intraneuronal inclusions of α-Syn, triggering a severe movement disorder beginning earliest at 8 months of age with weight loss, followed by a paresis of the hind limbs, finally leading to paralysis and death. In this study α-Syn^A53T^ mice were treated with 10 or 30 mg/kg bw Fasudil from day 50 on until death (Fig. 4a), and compared to untreated α-Syn^A53T^ mice as well as wt littermate controls regarding weight loss, motor and cognitive behavior.

α-Syn^A53T^ mice and wt controls were weighed weekly to monitor for weight changes over the experimental time course. Regarding their maximum weight, α-Syn^A53T^ mice were insignificantly lighter (~33 g) than wt controls (~35 g). After onset of clinical symptoms, α-Syn^A53T^ mice rapidly lost weight as previously described [16], being statistically significant at time of sacrifice as compared to wt controls (wt ctrl: 34.0 ± 1.68 g; A53T ctrl: 26.7 ± 0.79 g, *p* = 0.003, *T*-Test; A53T Fas10: 27.7 ± 1.23 g; A53T Fas30: 26.1 ± 1.29 g). Fasudil treatment did not have any significant effect on the weight of α-Syn^A53T^ mice (Fig. 4b, c).

Blood analysis did not show any significant differences between Fasudil-treated and untreated animals. Leukocyte (LEU) counts were detected between 3.1 and 9.0 × 10^3^/μl, erythrocytes (ERY) between 7.35 and 12.6 × 10^6^/μl, and thrombocytes (THR) ranged between 388 and 2102 × 10^3^/μl. Hemoglobin (HGB) ranged from 9.1 to 17.0 g/dl and hematocrit (HCT) from 26.4 to 50.6 %. Biochemical analysis of renal and hepatic function parameters revealed maximum values between 0.15 and 0.21 mg/dl of creatinine (CREA), aspartate aminotransferase (AST) was measured between 53 and 312 U/l, alanine aminotransferase (ALT) maximum values ranged between 18 and 113 U/l, and gamma-glutamyl transferase (GGT) was below detection level of <4 U/l (Additional file 2: Figure S2).

### Rotarod performance of α-Syn^A53T^ mice is impaired with disease onset

α-Syn^A53T^ mice and wt controls were pre-trained on the rotarod to obtain a stable performance. Rotarod testing after acute Fasudil treatment did not show any significant effects (Additional file 3: Figure S3). Rotarod testing at 100 and 200 days of age showed a stable performance without any signs of impairment in α-Syn^A53T^ mice (data not shown). Beginning from day 220 on, mice were tested twice a week on the rotarod, to monitor motor impairment as indication of onset of the clinical symptoms described for this model [16] (Fig. 4a). All mice maintained a stable rotarod performance over time (Fig. 4d), until with the onset of clinical symptoms α-Syn^A53T^ animals displayed a massive rotarod failure as a clear marker for declining motor function. Fasudil treatment did not improve rotarod performance as compared to untreated α-Syn^A53T^ mice. Figure 4e shows means ± SEM of last rotarod performance before sacrifice (wt ctrl: 277 ± 8 s; A53T ctrl: 57 ± 13 s; A53T Fas10: 47 ± 10 s; A53T Fas30: 55 ± 14 s).

### Survival of α-Syn^A53T^ mice is not altered by Fasudil treatment

After the onset of clinical symptoms and when mice undercut 50 % of the initial rotarod performance, mice were assessed for abnormalities in gait (Catwalk gait analysis) and cognition (novel object recognition test). Due to the fast progression of the disease in this particular model, α-Syn^A53T^ mice were sacrificed after the last behavioral test when hind limb paresis was present, to prevent animals from suffering. With regard to Fasudil treatment, no significant differences in survival time between the groups were detected (A53T ctrl: 509 ± 37 d; A53T Fas10: 428 ± 31 d; A53T Fas30: 523 ± 41 d) (Fig. 4f).

### Catwalk gait analysis reveals motor improvement after Fasudil treatment

**Fig. 5:**
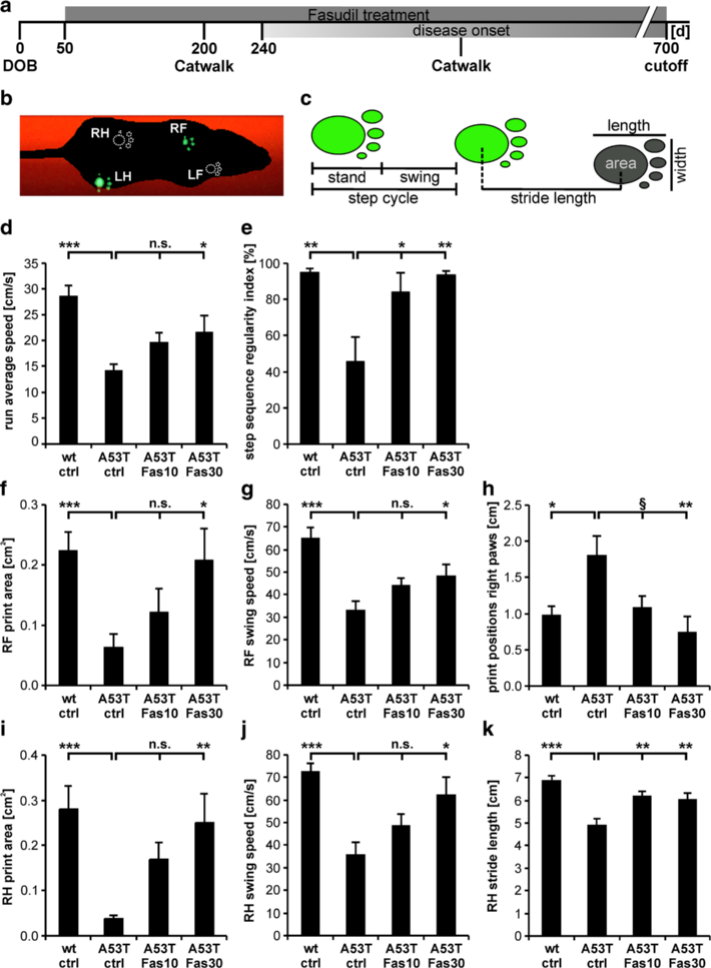
Catwalk gait analysis shows improved gait performance after Fasudil treatment in α-Syn^A53T^ mice. **a** α-Syn^A53T^ mice were treated with Fasudil at 10 mg/kg bw and 30 mg/kg bw starting at day 50 (DOB = day of birth). Catwalk gait analysis was performed after 200 days, and after the mouse showed clinical symptoms of the disease by impaired rotarod performance. **b** Footprints of mice were automatically detected by the Noldus Catwalk XT gait analysis system (RF = right front, LF = left front, RH = right hind, LH = left hind). **c** Different gait parameters like stand (duration in seconds of contact of a paw with the glass plate), swing (duration in seconds of no contact of a paw with the glass plate), step cycle, stride length, print area, print length and print width were analyzed. **d-k** Run average speed (**d**), step sequence regularity index as a fractional measure of inter-paw coordination (**e**), RF and RH print area (**f**, **i**), RF and RH swing speed (**g**, **j**), print positions of the right paws (**h**) and RH stride length (**k**) were significantly improved after Fasudil treatment as compared to untreated α-Syn^A53T^ controls. All data given as means ± SEM; n(wt ctrl) = 10, n(A53T ctrl) = 10, n(A53T Fas10) = 10, n(A53T Fas30) = 9; *p < 0.05, **p < 0.01, ***p < 0.001, ^§^ *p* = 0.06, n.s. = not significant; *T*-Test for comparisons between ctrl groups; ANOVA with Dunnett post-hoc test for multiple group comparisons between α-Syn^A53T^ groups

To detect more subtle differences in gait of α-Syn^A53T^ mice after treatment with Fasudil, mice were tested with the Catwalk gait analysis system, since this method is more sensitive than the rotarod [62]. α-Syn^A53T^ mice were tested after ‘disease onset’ as detected by rotarod failure (Fig. 5a). Footprints of the mice were automatically detected, and various gait parameters were analyzed (Fig. 5 b, c). Before disease onset at day 200, no significant differences between the groups were apparent (data not shown). Acute Fasudil treatment also did not have any significant effects in the multiparametric gait analysis (Additional file 4: Figure S4). After mice displayed clinical symptoms, long-term Fasudil treatment significantly improved gait performance of α-Syn^A53T^ mice as compared to untreated controls. In detail, run average speed was improved dose-dependently as compared to α-Syn^A53T^ control, with high Fasudil dosage reaching significance (wt ctrl: 28.6 ± 2.11 cm/s; A53T ctrl: 13.6 ± 1.20 cm/s, *p* = 7.05x10^-6^, *T*-test; A53T Fas10: 19.7 ± 1.85 cm/s; A53T Fas30: 21.7 ± 3.24 cm/s, *p* = 0.03, ANOVA with Dunnett post-hoc test; Fig. 5d). Step sequence regularity index (a fractional measure of inter-paw coordination) was significantly improved dose-dependently after Fasudil treatment (wt ctrl: 95.0 ± 2.13 %; A53T ctrl: 45.5 ± 13.59 %, *p* = 0.004, *T*-test; A53T Fas10: 83.9 ± 10.74 %, *p* = 0.03; A53T Fas30: 93.7 ± 2.10 %, *p* = 0.006, ANOVA with Dunnett post-hoc test; Fig. 5e). Print area, swing speed, stride length as well as print positions (distance between the position of the hind paw and the position of the previously placed front paw on the same side of the body and in the same step cycle) were significantly improved by high Fasudil treatment. As examples only right paws are displayed: right front (RF) print area was almost rescued as compared to controls (wt ctrl: 0.22 ± 0.03 cm^2^; A53T ctrl: 0.06 ± 0.02 cm^2^, *p* = 0.0003, *T*-test; A53T Fas10: 0.12 ± 0.03 cm^2^; A53T Fas30: 0.20 ± 0.05 cm^2^, *p* = 0.023, ANOVA with Dunnett post-hoc test; Fig. 5f), same effect was seen for right hind (RH) print area (wt ctrl: 0.28 ± 0.05 cm^2^; A53T ctrl: 0.03 ± 0.01 cm^2^, *p* = 0.0003, *T*-test; A53T Fas10: 0.16 ± 0.04 cm^2^; A53T Fas30: 0.25 ± 0.06 cm^2^, *p* = 0.009, ANOVA with Dunnett post-hoc test; Fig. 5i). Swing speed of the right front leg was significantly improved (wt ctrl: 64.9 ± 4.81 cm/s; A53T ctrl: 33.3 ± 3.80 cm/s, *p* = 4.2x10^-5^, *T*-test; A53T Fas10: 44.3 ± 3.04 cm/s; A53T Fas30: 48.3 ± 5.28 cm/s, *p* = 0.03, ANOVA with Dunnett post-hoc test; Fig. 5g), as was the swing speed for the right hind leg (wt ctrl: 72.5 ± 3.64 cm/s; A53T ctrl: 35.9 ± 5.53 cm/s, *p* = 0.0002, *T*-test; A53T Fas10: 48.6 ± 5.03 cm/s; A53T Fas30: 62.4 ± 7.61 cm/s, *p* = 0.014, ANOVA with Dunnett post-hoc test; Fig. 5j). Print positions of the right paws were significantly improved after high Fasudil treatment (wt ctrl: 0.97 ± 0.13 cm; A53T ctrl: 1.80 ± 0.27 cm, *p* = 0.019, *T*-test; A53T Fas10: 1.10 ± 0.16 cm, *p* = 0.06; A53T Fas30: 0.73 ± 0.23 cm, *p* = 0.005, ANOVA with Dunnett post-hoc test; Fig. 5h). Stride length of the right hind leg was significantly improved after Fasudil treatment as compared to untreated control (wt ctrl: 6.9 ± 0.21 cm; A53T ctrl: 4.9 ± 0.27 cm, *p* = 6.2x10^-5^, *T*-test; A53T Fas10: 6.2 ± 0.23 cm, *p* = 0.005; A53T Fas30: 6.0 ± 0.26 cm, *p* = 0.009, ANOVA with Dunnett post-hoc test; Fig. 5k). Taken together, Catwalk analysis revealed a dose-dependent, significant improvement of several gait parameters of α-Syn^A53T^ mice treated with Fasudil in a dose-dependent manner as compared to untreated α-Syn^A53T^ controls.

### NOR test shows rescue of cognitive impairment after Fasudil treatment

To test if Fasudil treatment influences cognition in α-Syn^A53T^ mice, the NOR-test was performed. Parameters of general activity (mobility, distance moved, mean velocity) were recorded, and the discrimination ratio between interaction with novel and familiar object was calculated as described before [6]. Before the first signs of clinical symptoms, mice were tested on day 200. Discrimination ratio showed no significant differences between the treatment groups at this time point (wt ctrl: 0.58 ± 0.05; A53T ctrl: 0.59 ± 0.05; A53T Fas10: 0.52 ± 0.04 d; A53T Fas30: 0.58 ± 0.07), neither did mobility (wt ctrl: 167 ± 18 s; A53T ctrl: 202 ± 15 s; A53T Fas10: 200 ± 9 s; A53T Fas30: 160 ± 14 s), moved distance (wt ctrl: 1117 ± 166 cm; A53T ctrl: 1677 ± 121 cm; A53T Fas10: 2146 ± 239 cm; A53T Fas30: 1850 ± 381 cm), and mean velocity (wt ctrl: 4.6 ± 0.69 cm/s; A53T ctrl: 5.7 ± 0.41 cm/s; A53T Fas10: 7.8 ± 0.77 cm/s; A53T Fas30: 6.2 ± 1.25 cm/s).

**Fig. 6:**
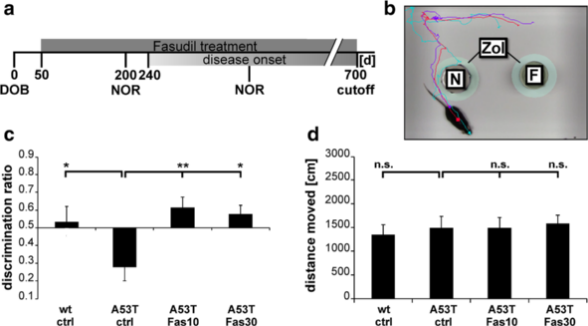
Fasudil treatment improves recognition memory of α-Syn^A53T^ mice. **a** α-Syn^A53T^ mice were treated with Fasudil at 10 mg/kg bw and 30 mg/kg bw starting at day 50 (DOB = day of birth). Novel object recognition test (NOR) was performed after 200 days, and after the mouse showed clinical symptoms of the disease by impaired rotarod performance. **b** Mice were videotaped in an arena with a familiar (F) and a novel (N) object. The time each mouse was heading the objects in the respective zone of interaction (ZOI) was recorded. General activity was tracked simultaneously. **c**, **d** The discrimination ratio (**c**) and distance moved (**d**) are displayed for the respective treatment groups as compared to controls after disease onset (n(wt ctrl) = 7, n(A53T ctrl) = 8, n(A53T Fas10) = 10, n(A53T Fas30) = 9). Data are displayed as means ± SEM; *p < 0.05, **p < 0.01; *T*-Test for comparisons between ctrl groups; ANOVA with Dunnett post-hoc test for multiple group comparisons between α-Syn^A53T^ groups

After the appearance of first clinical symptoms indicated by rotarod failure, mice were tested again in the NOR-test. Here, α-Syn^A53T^ ctrl mice showed a marked impairment in cognitive function compared to wt littermates, which was significantly reverted by Fasudil treatment (wt ctrl: 0.53 ± 0.09; A53T ctrl: 0.28 ± 0.08; *p* = 0.03, *T*-Test; A53T Fas10: 0.61 ± 0.06; A53T Fas30: 0.58 ± 0.05; *p* = 0.003, *p* = 0.010, ANOVA with Dunnett post-hoc test as compared to A53T ctrl) (Fig. 6c). Mobility time (wt ctrl: 139 ± 13 s; A53T ctrl: 113 ± 21 s; A53T Fas10: 129 ± 21 s; A53T Fas30: 116 ± 11 s), moved distance (wt ctrl: 1346 ± 214 cm; A53T ctrl: 1489 ± 249 cm; A53T Fas10: 1491 ± 216 cm; A53T Fas30: 1582 ± 175 cm) (Fig. 6d), as well as mean velocity (wt ctrl: 4.5 ± 0.71 cm/s; A53T ctrl: 5.0 ± 0.83 cm/s; A53T Fas10: 5.1 ± 0.73 cm/s; A53T Fas30: 5.3 ± 0.58 cm/s) were not significantly different between the groups.

### Analysis of α-Syn^A53T^ brain tissue depicts alterations in α-Syn pathology

**Fig. 7:**
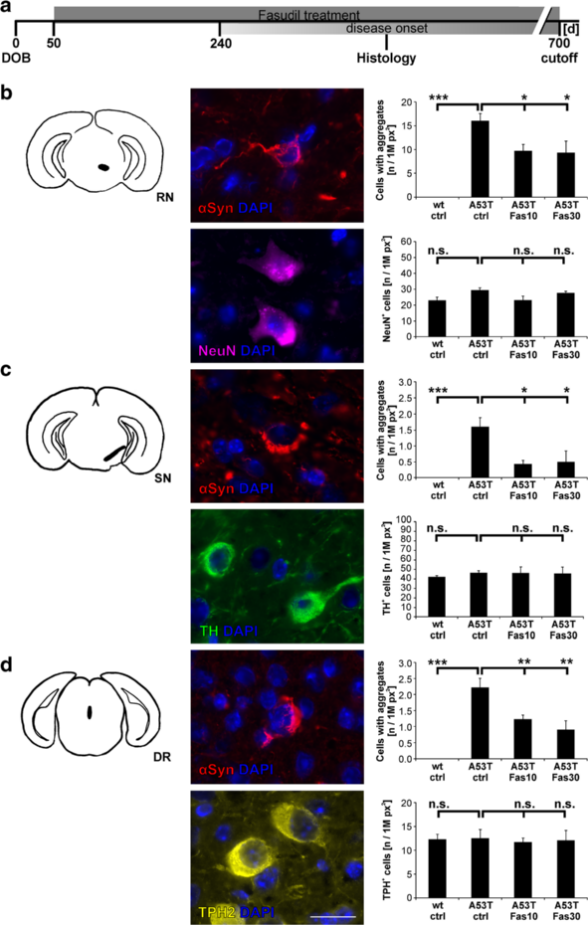
Fasudil treatment reduces the number of cells with α-Syn-aggregates without altering neuronal numbers in the midbrain of α-Syn^A53T^ mice. **a.** Immunohistochemical staining against proteinase-K resistant α-Syn aggregates, as well as NeuN, TH and TPH2 in combination with nuclear staining (DAPI) was performed to investigate the effect of Fasudil on α-Syn pathology in α-Syn^A53T^ mice after disease onset. Treatment groups were wildtype control (wt ctrl), α-Syn^A53T^ control (A53T ctrl), α-Syn^A53T^ with 10 mg/kg bw Fasudil (A53T Fas10) and α-Syn^A53T^ with 30 mg/kg bw Fasudil (A53T Fas30). **b.** Immunohistochemical analysis of red nucleus (RN; n(wt ctrl) = 5, n(A53T ctrl) = 7, n(A53T Fas10) = 6, n(A53T Fas30) = 6 for α-Syn aggregates; n(wt ctrl) = 6, n(A53T ctrl) = 6, n(A53T Fas10) = 6, n(A53T Fas30) = 6 for NeuN. **c.** Immunohistochemical analysis of substantia nigra (SN; n(wt ctrl) = 5, n(A53T ctrl) = 8, n(A53T Fas10) = 8, n(A53T Fas30) = 9 for α-Syn aggregates; n(wt ctrl) = 5, n(A53T ctrl) = 6, n(A53T Fas10) = 6, n(A53T Fas30) = 6 for TH. **d** Immunohistochemical analysis of the dorsal raphe nucleus (DR; n(wt ctrl) = 4, n(A53T ctrl) = 4, n(A53T Fas10) = 9, n(A53T Fas30) = 5 for α-Syn aggregates; n(wt ctrl) = 4, n(A53T ctrl) = 4, n(A53T Fas10) = 9, n(A53T Fas30) = 5 for TH. Drawings on the left show localization of respective brain regions, exemplary pictures in the middle show cells from respective brain regions with proteinase-K resistant α-Syn aggregates, as well as NeuN, TH, TPH2 and DAPI positive cells in brains of untreated α-Syn^A53T^ mice. Data on the right are presented as means ± SEM; * *p* <0.05; ** *p* < 0.01; *** *p* < 0.001; n.s. = not significant; *T*-Test for comparisons between untreated groups; ANOVA with Dunnett post-hoc test for multiple group comparisons between α-SynA53T groups; 1M px^2 ^= one million square pixels. Scale bar = 20 μm

To investigate the effect of Fasudil treatment on histopathology in the brain of α-Syn^A53T^ mice, an immunohistochemical analysis was performed after 200 days as well as after disease onset (Fig. 7a). Cells showing α-Syn aggregates after proteinase-K digestion were quantified in three different brain regions, namely red nucleus (RN), substantia nigra pars compacta (SN) and dorsal raphe nucleus (DR), and compared between the different treatment groups and wt littermate controls. After 200 days no proteinase-K resistant α-Syn aggregates could be detected in α-Syn^A53T^ mice (data not shown). As expected, no α-Syn pathology was detected in the wt controls, whereas at disease-onset α-Syn^A53T^ mice showed α-Syn aggregates in all investigated brain regions. Interestingly, Fasudil treatment significantly decreased the number of cells showing α-Syn aggregates.

In the RN, cells with α-Syn aggregates were reduced by about 40 % (A53T Fas10: 9.68 ± 1.42 cells/1 M px^2^, *p* = 0.045; A53T Fas30: 9.32 ± 2.45 cells/1 M px^2^, *p* = 0.034, ANOVA with Dunnett post-hoc test, Fig. 7b) as compared to vehicle-treated α-Syn^A53T^ mice (A53T ctrl: 16.04 ± 1.54 cells/1 M px^2^). Importantly, the evaluation of total neuronal numbers by NeuN-staining in the RN did not reveal any significant differences between the treatment groups (wt ctrl: 22.97 ± 2.37 cells/1 M px^2^, A53T ctrl: 29.22 ± 1.70 cells/1 M px^2^, A53T Fas10: 23.07 ± 2.99 cells/1 M px^2^, A53T Fas30: 27.64 ± 1.08 cells/1 M px^2^, n.s., ANOVA, Fig. 7b).

In the SN the Fasudil treatment reduced cells with α-Syn aggregates by more than 70 % as compared to vehicle-treated animals (A53T ctrl: 1.60 ± 0.28 cells/1 M px^2^; A53T Fas10: 0.41 ± 0.13 cells/1 M px^2^, *p* = 0.014; A53T Fas30: 0.50 ± 0.34 cells/1 M px^2^, *p* = 0.019, ANOVA with Dunnett post-hoc test, Fig. 7c). The evaluation of absolute TH-positive cell numbers in the SN did not show any significant differences between the treatment groups (wt ctrl: 42.01 ± 1.83 cells/1 M px^2^, A53T ctrl: 46.21 ± 2.72 cells/1 M px^2^, A53T Fas10: 46.11 ± 7.25 cells/1 M px^2^, A53T Fas30: 45.43 ± 7.57 cells/1 M px^2^, n.s., ANOVA, Fig. 7c).

Fasudil treatment lead also to a reduction of cells bearing α-Syn aggregates in the DR by more than 50 % as compared to vehicle-treated controls (A53T: 2.22 ± 0.28 cells/1 M px^2^; A53T Fas10: 1.22 ± 0.13 cells/1 M px^2^, *p* = 0.009; A53T Fas30: 0.90 ± 0.27 cells/1 M px^2^, *p* = 0.002, ANOVA with Dunnett post-hoc test, Fig. 7d). The evaluation of total TPH2-positive cells in the DR did not reveal any significant differences (wt ctrl: 12.25 ± 1.12 cells/1 M px^2^; A53T ctrl: 12.43 ± 1.91 cells/1 M px^2^; A53T Fas10: 11.65 ± 0.88 cells/1 M px^2^; A53T Fas30: 12.03 ± 2.11 cells/1 M px^2^, n.s., ANOVA, Fig. 7d).

## Discussion

α-Syn aggregation is a major pathophysiological step in the development of PD. Since its discovery [41] and consequent identification as most abundant protein component in Lewy bodies [53], α-Syn was shown to be involved in numerous cellular processes [4, 14, 29, 66]. Therapeutic approaches addressing the aggregation of α-Syn thus hold the promise to result in true disease-modification and attenuate progression in PD.

Recently, ROCK was characterized as promising new molecular therapeutic target. Its pharmacological inhibition resulted in prevention of neurodegeneration, fostered axonal regeneration and beneficially regulated microglial dysfunction in models of PD and other neurodegenerative disorders [3, 8, 26, 27, 50, 58–61, 63]. Here, we evaluated the anti-aggregative potential of pharmacological ROCK inhibition using Fasudil, which is already approved for clinical use in humans [44].

In contrast to the structurally unrelated 4-aminopyridine derivative Y-27632, Fasudil treatment significantly reduced the number of H4 cells with SYPH1/synT inclusions *in vitro*, which was confirmed by SEC-HPLC followed by dot-blot analysis. To understand the molecular basis of this reduced aggregate formation, we performed a cell-free thioflavin-T aggregation assay. Interestingly, again in contrast to Y-27632, Fasudil significantly delayed α-Syn aggregation, which was affirmed by electron microscopy and indicated a direct inhibiting function of Fasudil on α-Syn aggregation. Since this effect was observed in a cell-free assay, it was not mediated by the ROCK-inhibiting properties of Fasudil. NMR spectroscopy revealed a direct binding of Fasudil to two tyrosine residues in the C-terminal region of α-Syn, namely Y133 and Y136, and site-directed mutagenesis of these residues confirmed the biological relevance of this interaction in the H4 aggregation model *in vitro*. The C-terminal region was recently shown to be involved in α-Syn fibrillization [25], with C-terminal truncation accelerating the ability of α-Syn to form fibrils *in vitro* [32] and *in vivo* [35, 37], whereas negative charge in the C-terminus of full-length α-Syn delayed fibril formation [23, 25]. Interestingly, Izawa and colleagues suggested Y136 as critical element in promoting fibril formation [25]. Our data now show that Fasudil binds to precisely this particular C-terminal tyrosine. This data prompted us to assess the effects of Fasudil treatment in an *in vivo*-model of α-Syn aggregation.

The influence of long-term Fasudil treatment on α-Syn pathology *in vivo* was investigated in the α-Syn^A53T^ mouse model [16], corresponding to a mutation found in a familial form of PD [47]. Animals were treated with Fasudil from day 50 on until death, and no acute Fasudil effects with regard to motor behavior were detected. The average survival time of α-Syn^A53T^ mice with high Fasudil treatment was more than 500 days, and did not differ significantly from untreated α-Syn^A53T^ mice. Fasudil has already been used in animal models of neurodegeneration and was well tolerated in treatment paradigms for up to four months [58–60]. Here we present the first long-term animal study supporting the favorable safety profile of Fasudil over more than 15 months average treatment time, without any obvious unwanted side effects. Importantly, blood analysis revealed no significant changes after Fasudil treatment, with all parameters being in the normal range for laboratory mice [7, 9, 11, 49]. Disease onset could be clearly determined by rotarod failure, which is in line with previous findings [16]. No rotarod failure of α-Syn^A53T^ mice was detected before disease onset, although recent studies report contradictory findings in the same model [19, 45, 68].

To further investigate motor performance, the Catwalk gait analysis system was used. In contrast to the rotarod, here the animals walk freely and are not forced due to an automated treadmill, which allows a highly sensitive detection of subtle gait changes [62]. α-Syn^A53T^ mice showed a massive decline in stride length and velocity at disease onset, which could be translated to gait deficits observed in PD patients [33, 54] and is in line with previous findings in this model [17, 45]. Interestingly, we show that numerous gait parameters were significantly improved by long-term Fasudil treatment in a dose-dependent manner.

Since Fasudil attenuated aggregation of α-Syn *in vitro*, we analyzed whether its application in the α-Syn^A53T^ mouse model also results in a histological improvement *in vivo*. After 200 days no α-Syn pathology could be detected in α-Syn^A53T^ mice, which is in line with previous findings in this model [16]. Remarkably, the amount of neurons in the RN and SN showing α-Syn pathology after proteinase-K digestion at disease-onset was significantly decreased after Fasudil treatment, whereas the number of NeuN-positive cells in the RN was not affected. The number of TH-positive cells in the SN was also not affected, which is in line with previous findings showing that TH neurons in the SN of α-Syn^A53T^ mice do not display the same selective vulnerability as in humans [16]. Since RN and SN are highly involved in motor function and coordination in rodents, increased α-Syn pathology in these regions was suggested to compromise their function and thus contribute to the motor deficits [16]. We hypothesize that specific binding of Fasudil to α-Syn, as demonstrated by NMR analysis, contributes to the reduction of α-Syn pathology in RN and SN, thereby improving fine motor function.

In addition to motor deficits, PD patients show numerous non-motor symptoms, including cognitive decline [5, 10]. We therefore analyzed the presence of cognitive impairments and the putative therapeutic effect of Fasudil in α-Syn^A53T^ mice. The NOR-test has been extensively used to detect cognitive deficits in numerous disease models [6, 20, 39]. In contrast to a previous study in a synucleinopathy mouse model [40], we did not detect any significant differences in cognitive behavior after 200 days in our α-Syn^A53T^ mice. However, at onset of motor symptoms there was a significant alteration in the novelty seeking behavior, which was reversed by Fasudil treatment. This is in line with previous findings showing significant deficits in short term memory in this mouse line, postulated to be mediated by a dysfunctional dopamine system [36, 45, 51, 67]. In addition to dopaminergic projections, other neurotransmitter systems are known to contribute to motor and particularly non-motor symptoms in PD. For example, the serotonergic system is involved in memory function in PD [21]. The reduction of α-Syn pathology in the DR together with the recovery of recognition memory by Fasudil suggests that drugs acting in a disease-modifying manner by modification of α-Syn aggregation may also successfully target non-motor symptoms induced by non-dopaminergic pathology.

In regard to transfer of these findings into clinical trials, Fasudil has a highly promising translational potential, as it is already approved for clinical use in humans in Japan, showing a very favorable safety profile [44]. Fasudil is active after oral administration and is capable of crossing the blood–brain-barrier [56, 60]. Although other pharmacological ROCK inhibitors, e.g. Y-27632, are more selective towards ROCK, the latter compound did not affect α-Syn aggregation. Interestingly, Y-27632 was shown to improve degradation of mutant huntingtin as well as motor performance in the R6/2 mouse model of Huntington’s disease [34]. Besides, the effect of NSAIDs on lowering the amount of Aβ42 in models of Alzheimer’s disease is mediated by ROCK inhibition and can be mimicked by Y-27632 [69]. Thus, in addition to the specific anti-aggregative effect of Fasudil through direct molecular interaction with α-Syn, ROCK inhibition may also contribute to a depletion of aggregation-prone proteins. Increased levels of ROCK were also recently discovered in brains of patients with progressive supranuclear palsy and corticobasal degeneration, and ROCK inhibition attenuated tau pathology in *Drosophila* [15]. Thus, different lines of evidence suggest that ROCK is involved in the pathogenesis of aggregation disorders and that ROCK inhibition represents a promising therapeutic strategy [50, 58–61].

Here we describe for the first time to our knowledge the anti-aggregative potential of the ROCK-inhibitor Fasudil via direct binding to α-Syn, which suggests a new treatment approach employing a double function of Fasudil. Although in recent years a number of α-Syn aggregation inhibitors have been described [1, 13, 55, 57], most of them are “pan-assay interference compounds” with a questionable clinical perspective [2]. High-throughput screening revealed more specific compounds like the α-Syn-oligomer-binding Anle-138b [65] or recently discovered monomeric α-Syn-binding BIOD303 [43], appearing more auspicious. One of the major obstacles of these new compounds, however, is the so far missing approval for human treatment with largely unclear safety and tolerability issues. Since Fasudil is already approved for clinical human treatment, the repurposing of this drug for the treatment of PD and other synucleinopathies appears therefore highly promising.

## Conclusion

We describe here the anti-aggregative potential of the ROCK-inhibitor Fasudil via direct binding to α-Syn, which opens a new treatment avenue employing a ROCK-independent function of Fasudil in PD and other synucleinopathies. This is of particular interest, as Fasudil is already licensed in Japan for the treatment of subarachnoid haemorrhage-induced vasospasms and has been widely used with a beneficial safety profile. Since disease-modifying therapies for neurodegenerative diseases are urgently needed, the translation of our findings into a clinical pilot study appears realistic in the near future.

### Ethical approval

“All applicable international, national, and/or institutional guidelines for the care and use of animals were followed.”

## Additional files

Additional file 1: Figure S1. Y-27632 treatment has no effect on synT aggregation in H4 cells *in vitro*. **a** H4 neuroglioma cells were seeded in the presence or absence of Y-27632 and 24 h later transfected with plasmids encoding for synT and SYPH1. 24 h after transfection, cultures were investigated by immunocytochemistry (ICC). **b** ICC of synT and SYPH1 in H4 cells 24 h after transfection, treated with 5 or 20 μM Y-27632. Scale bar: 50 μm. **c** Quantification of transfected H4 cells with inclusions 24 h after transfection and treatment with different Y-27632 concentrations. n.s. = not significant, ANOVA, *n* = 3. Data are given as mean ± SEM, n.s. = not significant, ANOVA. (TIF 723 kb) Additional file 2: Figure S2. Blood cell counts and biochemical analysis of renal and hepatic function parameters in α-Syn^A53T^ mice. **a** Leucocytes (LEU). **b** Erythrocytes (ERY). **c** Thrombocytes (THR). **d** Hemoglobin (HGB). **e** Hematocrit (HCT). **f** Creatinine (CREA). **g** Alanine aminotransferase (ALT). **h** Aspartate aminotransferase (AST). **i** gamma-glutamyl transferase (GGT, # = all values below detection level). All measured parameters were in the normal range for laboratory mice. **a-e**: n(wt ctrl) = 5, n(A53T ctrl) = 2, n(A53T Fas10) = 4, n(A53T Fas30) = 5; **f-i**: n(wt ctrl) = 5, n(A53T ctrl) = 1, n(A53T Fas10) = 8, n(A53T Fas30) = 7. (TIF 125 kb) Additional file 3: Figure S3. Acute Fasudil treatment does not affect rotarod performance of α-Syn^A53T^ mice. **a** α-Syn^A53T^ mice were tested on the rotarod before Fasudil treatment with 30 mg/kg bw for 24 h via the drinking water, as well as directly after treatment. **b** α-Syn^A53T^ mice were tested on the rotarod 24 h before Fasudil treatment with 20 mg/kg bw via oral gavage. 30 min after application mice were tested again on the rotarod. **c** No significant differences were detected after Fasudil treatment for 24 h via the drinking water as compared to controls. Data are given as means ± SEM; n(A53T ctrl) = 7, n(A53T Fas) = 7; n.s. = not significant; *T*-Test. **d** No significant differences were detected 30 min after Fasudil treatment via oral gavage as compared to controls. Data are given as means ± SEM; n(A53T ctrl) = 9, n(A53T Fas) = 9; n.s. = not significant; *T*-Test. (TIF 176 kb) Additional file 4: Figure S4. Acute Fasudil treatment does not affect Catwalk performance of α-Syn^A53T^ mice. **a** α-Syn^A53T^ mice were tested on the Catwalk before Fasudil treatment with 30 mg/kg bw for 24 h via the drinking water, as well as directly after treatment. **b** α-Syn^A53T^ mice were tested on the Catwalk 24 h before Fasudil treatment with 20 mg/kg bw via oral gavage. 30 min after application mice were tested again on the Catwalk. **c, e, g, i** No significant differences were detected after Fasudil treatment for 24 h via the drinking water as compared to controls regarding run average speed (**c**), RH print area (**e**), RH swing speed (**g**), and RH stride length (**i**). Data are given as means ± SEM; n(A53T ctrl) = 7, n(A53T Fas) = 7; n.s. = not significant; *T*-Test. **d, f, h, j** No significant differences were detected 30 min after Fasudil treatment via oral gavage as compared to controls regarding run average speed (**d**), RH print area (**f**), RH swing speed (**h**), and RH stride length (**j**). Data are given as means ± SEM; n(A53T ctrl) = 9, n(A53T Fas) = 9; n.s. = not significant; *T*-Test. (TIF 435 kb)
