# Supplementary figures and images for: Fasudil attenuates aggregation of α-synuclein in models of Parkinson’s disease
Source: Acta Neuropathol Commun. 2016 Apr 22;4:39. doi: 10.1186/s40478-016-0310-y (PMC4840958; doi:10.1186/s40478-016-0310-y)

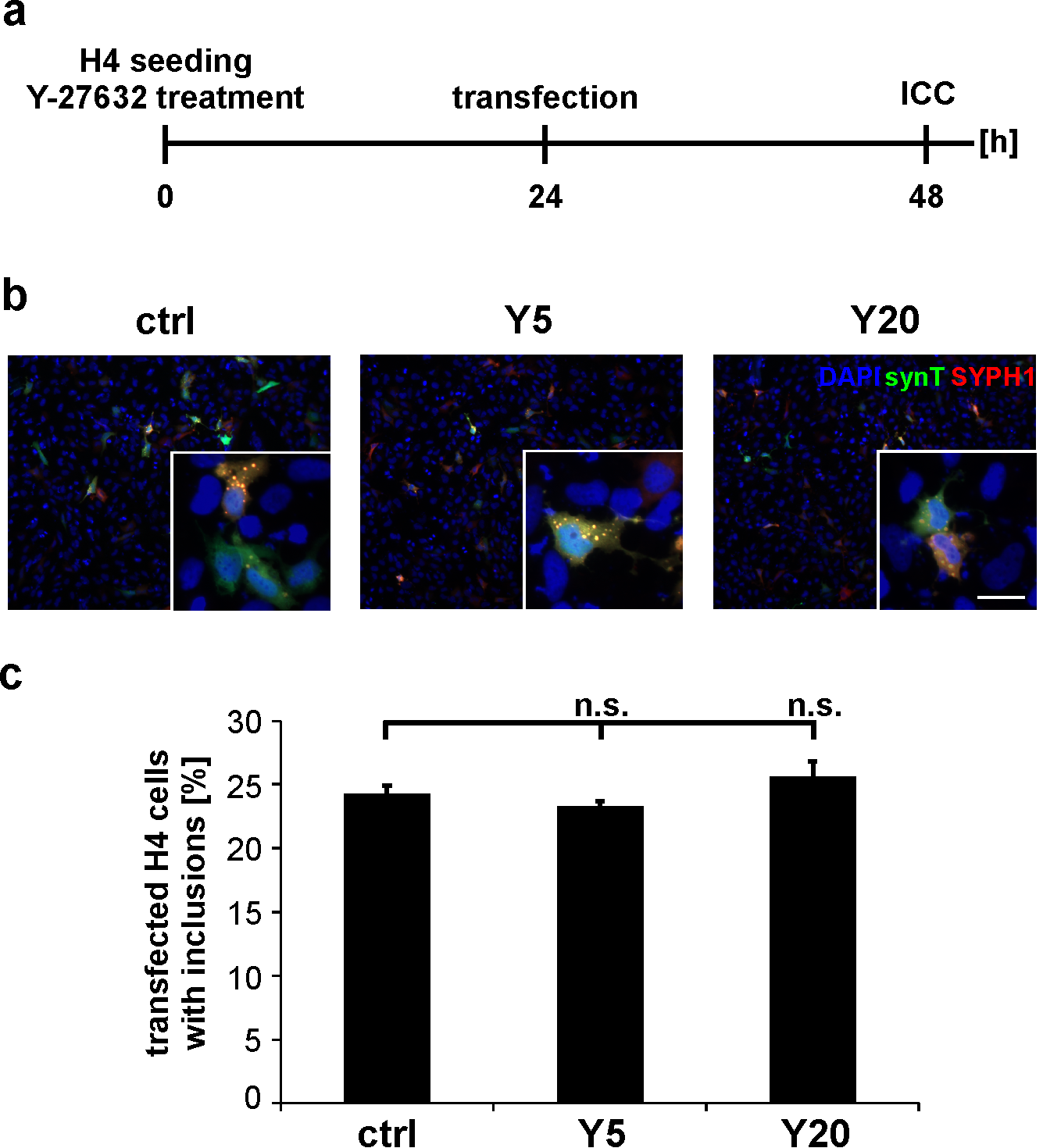

Supplement: Additional file 1: Figure S1. — Y-27632 treatment has no effect on synT aggregation in H4 cells in vitro. a H4 neuroglioma cells were seeded in the presence or absence of Y-27632 and 24 h later transfected with plasmids encoding for synT and SYPH1. 24 h after transfection, cultures were investigated by immunocytochemistry (ICC). b ICC of synT and SYPH1 in H4 cells 24 h after transfection, treated with 5 or 20 μM Y-27632. Scale bar: 50 μm. c Quantification of transfected H4 cells with inclusions 24 h after transfection and treatment with different Y-27632 concentrations. n.s. = not significant, ANOVA, n = 3. Data are given as mean ± SEM, n.s. = not significant, ANOVA. (TIF 723 kb) [file 40478_2016_310_MOESM1_ESM.tif]

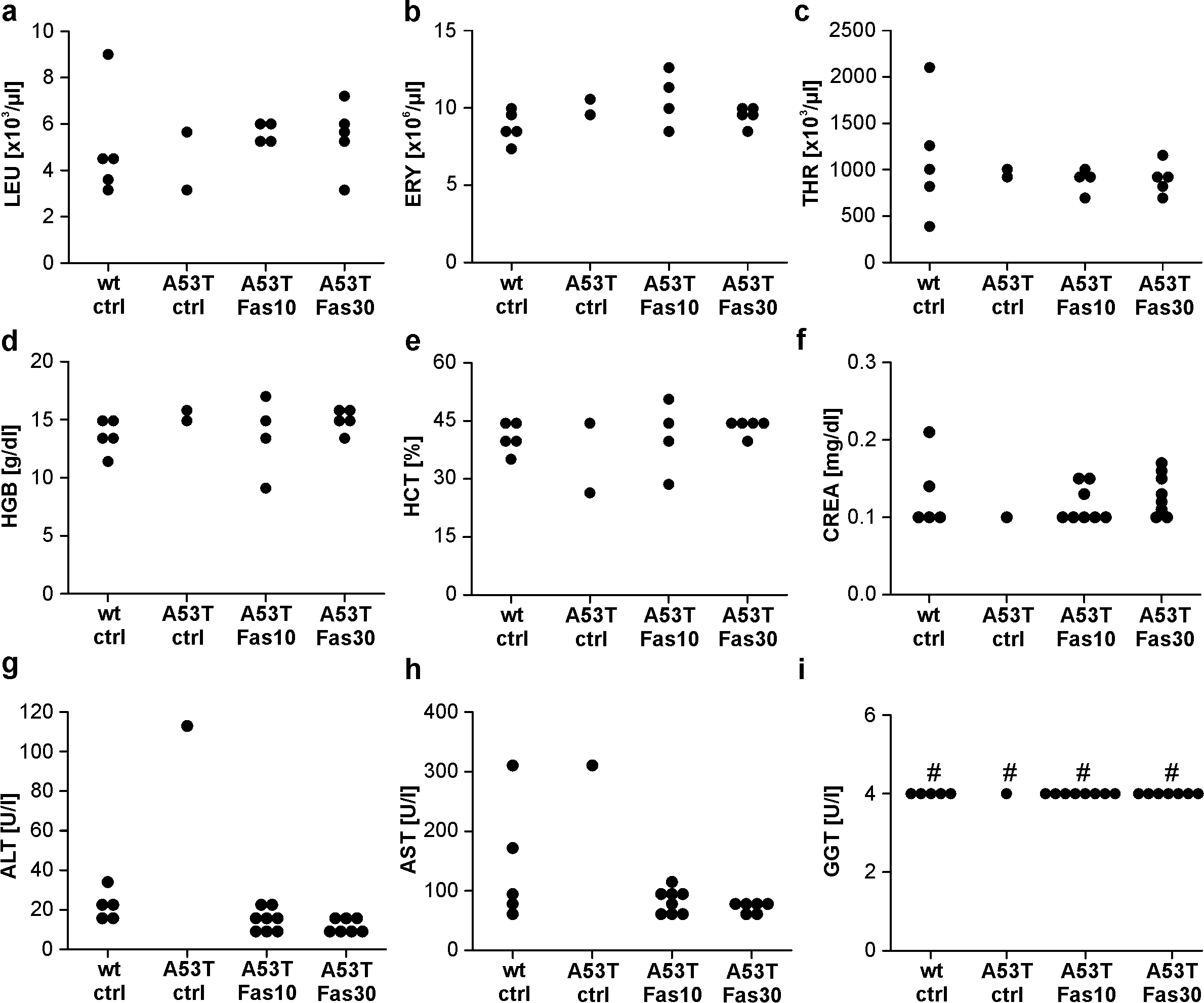

Supplement: Additional file 2: Figure S2. — Blood cell counts and biochemical analysis of renal and hepatic function parameters in α-SynA53T mice. a Leucocytes (LEU). b Erythrocytes (ERY). c Thrombocytes (THR). d Hemoglobin (HGB). e Hematocrit (HCT). f Creatinine (CREA). g Alanine aminotransferase (ALT). h Aspartate aminotransferase (AST). i gamma-glutamyl transferase (GGT, # = all values below detection level). All measured parameters were in the normal range for laboratory mice. a-e: n(wt ctrl) = 5, n(A53T ctrl) = 2, n(A53T Fas10) = 4, n(A53T Fas30) = 5; f-i: n(wt ctrl) = 5, n(A53T ctrl) = 1, n(A53T Fas10) = 8, n(A53T Fas30) = 7. (TIF 125 kb) [file 40478_2016_310_MOESM2_ESM.tif]

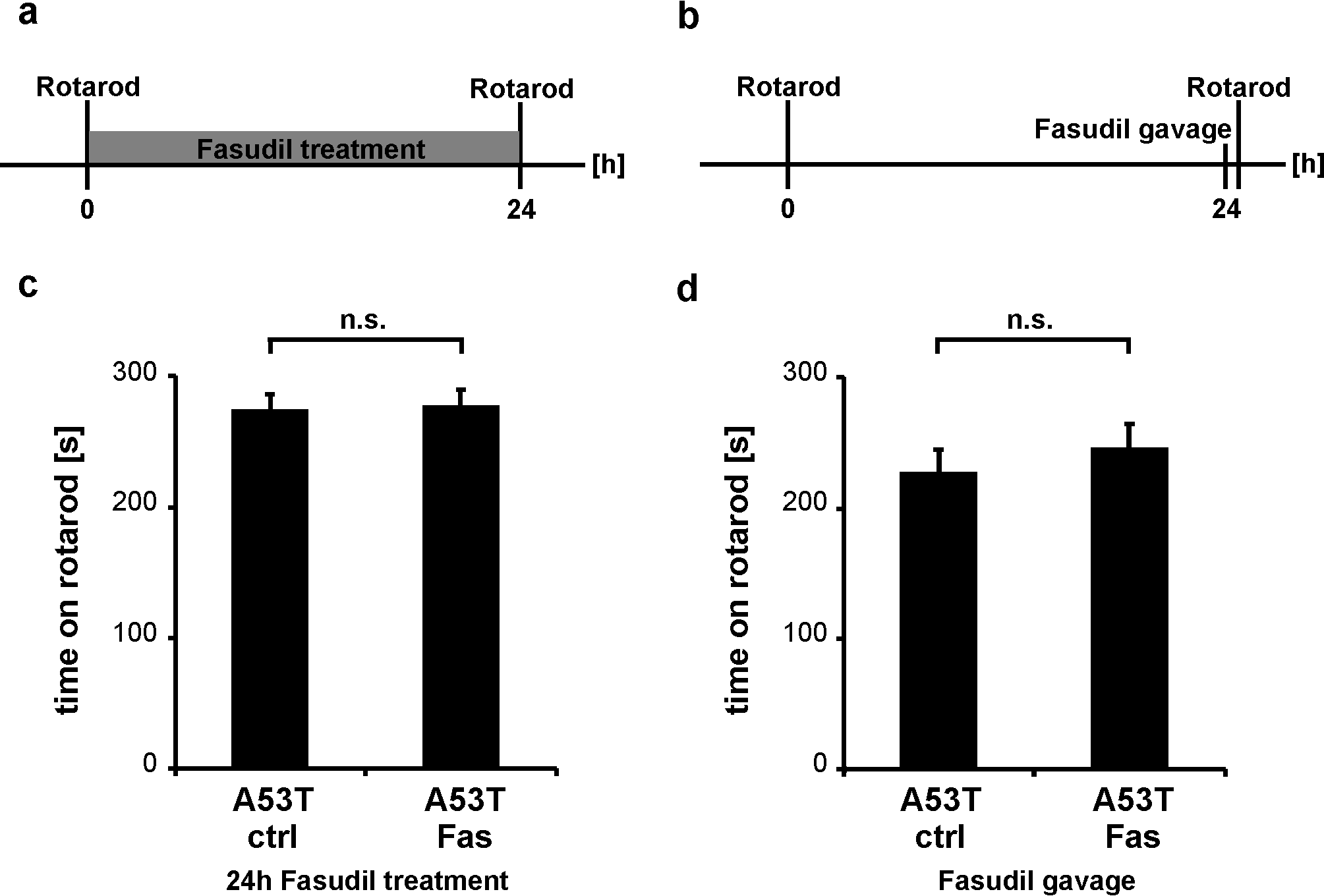

Supplement: Additional file 3: Figure S3. — Acute Fasudil treatment does not affect rotarod performance of α-SynA53T mice. a α-SynA53T mice were tested on the rotarod before Fasudil treatment with 30 mg/kg bw for 24 h via the drinking water, as well as directly after treatment. b α-SynA53T mice were tested on the rotarod 24 h before Fasudil treatment with 20 mg/kg bw via oral gavage. 30 min after application mice were tested again on the rotarod. c No significant differences were detected after Fasudil treatment for 24 h via the drinking water as compared to controls. Data are given as means ± SEM; n(A53T ctrl) = 7, n(A53T Fas) = 7; n.s. = not significant; T-Test. d No significant differences were detected 30 min after Fasudil treatment via oral gavage as compared to controls. Data are given as means ± SEM; n(A53T ctrl) = 9, n(A53T Fas) = 9; n.s. = not significant; T-Test. (TIF 176 kb) [file 40478_2016_310_MOESM3_ESM.tif]

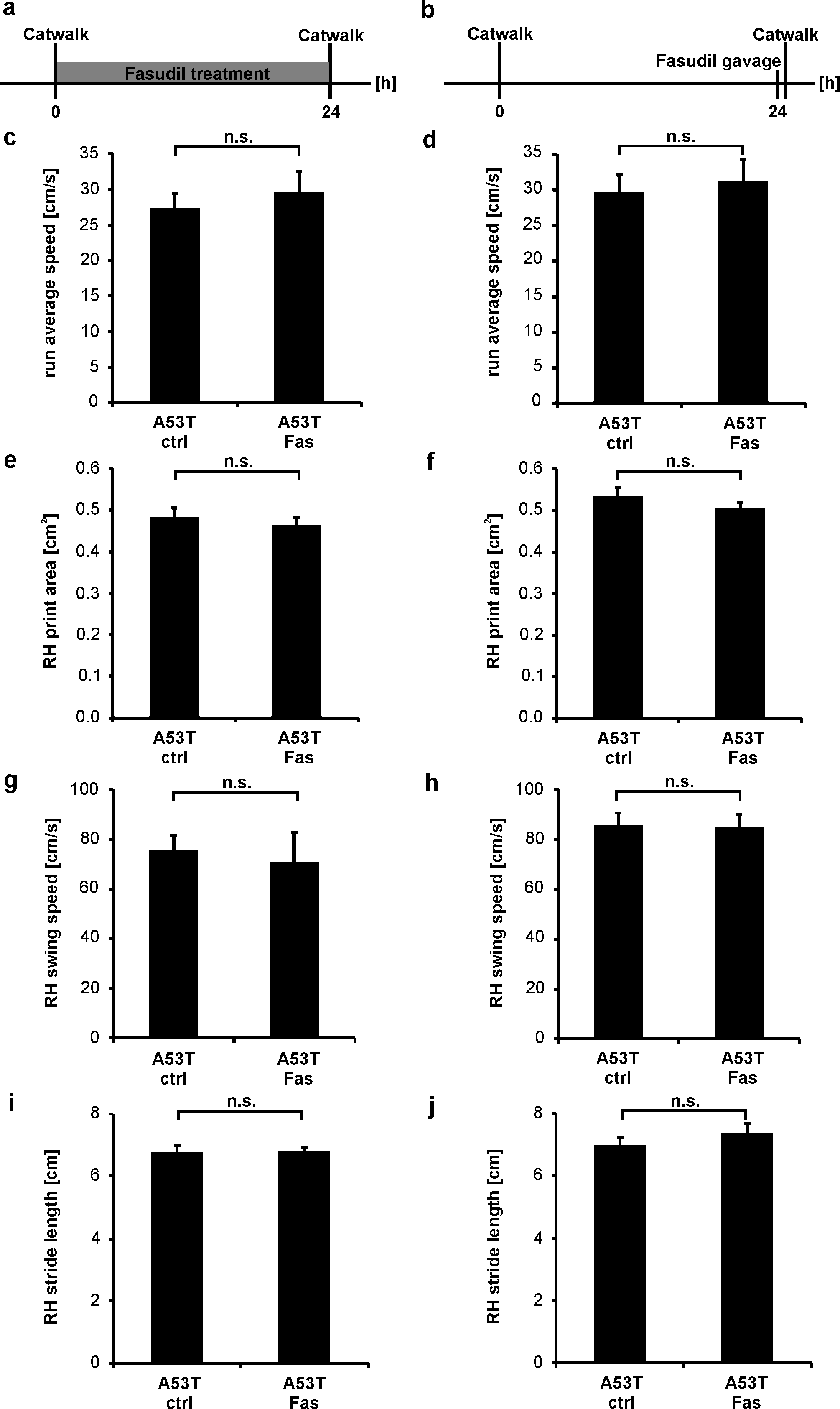

Supplement: Additional file 4: Figure S4. — Acute Fasudil treatment does not affect Catwalk performance of α-SynA53T mice. a α-SynA53T mice were tested on the Catwalk before Fasudil treatment with 30 mg/kg bw for 24 h via the drinking water, as well as directly after treatment. b α-SynA53T mice were tested on the Catwalk 24 h before Fasudil treatment with 20 mg/kg bw via oral gavage. 30 min after application mice were tested again on the Catwalk. c, e, g, i No significant differences were detected after Fasudil treatment for 24 h via the drinking water as compared to controls regarding run average speed (c), RH print area (e), RH swing speed (g), and RH stride length (i). Data are given as means ± SEM; n(A53T ctrl) = 7, n(A53T Fas) = 7; n.s. = not significant; T-Test. d, f, h, j No significant differences were detected 30 min after Fasudil treatment via oral gavage as compared to controls regarding run average speed (d), RH print area (f), RH swing speed (h), and RH stride length (j). Data are given as means ± SEM; n(A53T ctrl) = 9, n(A53T Fas) = 9; n.s. = not significant; T-Test. (TIF 435 kb) [file 40478_2016_310_MOESM4_ESM.tif]
